# Spotting priming-active compounds using parsley cell cultures in microtiter plates

**DOI:** 10.1186/s12870-023-04043-y

**Published:** 2023-02-02

**Authors:** Kyra Hoffmann, Jana Viola Schilling, Georg Wandrey, Tim Welters, Stefan Mahr, Uwe Conrath, Jochen Büchs

**Affiliations:** 1 AVT – Biochemical Engineering, RWTH Aachen University, 51 Forckenbeckstr, 52074 Aachen, Germany; 2 Department of Plant Physiology, RWTH Aachen University, 1 Worringer Weg, 52074 Aachen, Germany

**Keywords:** Defense priming, Parsley cell culture, Salicylic acid, Pep13, Furanocoumarins, High-throughput, Scale-down, Online monitoring, Fluorescence monitoring, Oxygen transfer rate (OTR)

## Abstract

**Background:**

Conventional crop protection has major drawbacks, such as developing pest and pathogen insensitivity to pesticides and low environmental compatibility. Therefore, alternative crop protection strategies are needed. One promising approach treats crops with chemical compounds that induce the primed state of enhanced defense. However, identifying priming compounds is often tedious as it requires offline sampling and analysis. High throughput screening methods for the analysis of priming-active compounds have great potential to simplify the search for such compounds. One established method to identify priming makes use of parsley cell cultures. This method relies on measurement of fluorescence of furanocoumarins in the final sample. This study demonstrates for the first time the online measurement of furanocoumarins in microtiter plates. As not all plants produce fluorescence molecules as immune response, a signal, which is not restricted to a specific plant is required, to extend online screening methods to other plant cell cultures. It was shown that the breathing activity of primed parsley cell cultures increases, compared to unprimed parsley cell cultures. The breathing activity can by monitored online. Therefore, online identification of priming-inducing compounds by recording breathing activity represents a promising, straight-forward and highly informative approach. However, so far breathing has been recorded in shake flasks which suffer from low throughput. For industrial application we here report a high-throughput, online identification method for identifying priming-inducing chemistry.

**Results:**

This study describes the development of a high-throughput screening system that enables identifying and analyzing the impact of defense priming-inducing compounds in microtiter plates. This screening system relies on the breathing activity of parsley cell cultures. The validity of measuring the breathing activity in microtiter plates to drawing conclusions regarding priming-inducing activity was demonstrated. Furthermore, for the first time, the fluorescence of the priming-active reference compound salicylic acid and of furanocoumarins were simultaneously monitored online. Dose and time studies with salicylic acid-treated parsley cell suspensions revealed a wide range of possible addition times and concentrations that cause priming. The online fluorescence measuring method was further confirmed with three additional compounds with known priming-causing activity.

**Conclusions:**

Determining the OTR, fluorescence of the priming-active chemical compound SA and of furanocoumarins in parsley suspension cultures in MTPs by online measurement is a powerful and high-throughput tool to study possible priming compounds. It allows an in-depth screening for priming compounds and a better understanding of the priming process induced by a given substance. Evaluation of priming phenomena via OTR should also be applicable to cell suspensions of other plant species and varieties and allow screening for priming-inducing chemical compounds in intact plants. These online fluorescence methods to measure the breathing activity, furanocoumarin and SA have the potential to accelerate the search for new priming compounds and promote priming as a promising, eco-friendly crop protection strategy.

**Supplementary Information:**

The online version contains supplementary material available at 10.1186/s12870-023-04043-y.

## Background

Intensive agriculture depends on the heavy use of synthetic pesticides. The long-term prognosis for their application is unfavorable, as resistance formation of pests and pathogens and the associated loss of biodiversity is just one consequence [1–3]. Hence, the pressure from consumers and politics on agriculture and pesticide producers to practice sustainable cultivation and plant protection increases. The natural defense of plants is based on plant defense compounds such as phytoalexins that accumulate in response to pathogen contact. However, from the onset of pathogen infestation and following defense response, high fitness losses often occur on the infected part of the plant. The defense response becomes faster and more efficient, when the plant is in an increased state of alert. One type of induced resistance resulting in an increased state of alert is the systemic acquired resistance (SAR). SAR can also be induced with non-pathogenic chemical compounds, such as salicylic acid (SA) [4–6]. Defense priming enables plants to resist biotic and abiotic stresses more quickly and with fewer fitness costs [4, 5, 7–9]. Thus, one approach for sustainable agriculture is using substances that induce defense priming. In 1992, a screening system to detect priming-inducing chemistry in parsley cell cultures has been introduced [10]. The system is based on fluorescence measurements of furanocoumarins [11]. Parsley cell cultures were primed with a compound such as salicylic acid (SA) after 3 h—72 h cultivation time. After 96 h the elicitor Pep13 was added to the cell cultures, to stimulate an immune response. Pep13 is a 13 amino acid peptide of a glycoprotein from the soybean pathogen *phytophthora sojae* [12]. If priming occurred, cell cultures responded with increased production of furanocoumarins, fluorescent phytochemicals that belong to the phytoalexins. Furanocoumarins were qualitatively detected by fluorescence measurement at an excitation wavelength of 335 nm and an emission wavelength of 398 nm [10, 11, 13, 14]. Studies based on this method of investigating priming, are discussed below.

One online screening technique reported for parsley cell cultures is based on applying the respiratory activity monitoring system (RAMOS) [15, 16]. RAMOS measures the OTR to identify putative priming compounds in shake flasks [17]. To establish the online screening system, parsley cell cultures were pretreated with compounds that were known to be active, or inactive, at activating defense priming before they were treated with Pep13 [10, 11, 13]. Parsley cell cultures were demonstrated to respond with an increase in the OTR after priming and subsequent stimulation with an elicitor [17]. The increase of the OTR after the addition of the elicitor Pep13 was higher, if the cell cultures were previously primed with SA or e.g. the known priming-active SA derivative 4-chlorosalicylic acid (4-CSA). The treatment with the known priming-inactive compounds 4-hydroxybenzoic acid (4-HBA) and 3-hydroxybenzoic acid (3-HBA) did not lead to an increase in the OTR [17]. From the course of the OTR, it was possible to infer which compound was active at priming of the cell cultures [17]. As the OTR is an unspecific signal, applicable for all plant suspension cultures, this technique bears the potential to be transferred to other plant cell cultures.

Another innovative screening devise for response-screening activity in plant cells is based on the online measurement of the defense-associated plant hormone ethylene [18]. In this approach, cultured parsley cells were grown in a RAMOS system equipped with electrochemical ethylene sensors. The devise enables the simultaneous recording of the OTR and ethylene. It thus provides more information on the complex interplay of signaling different signaling compounds in a given plant species.

A recently published study aimed at transferring the RAMOS technique from cell suspension cultures to *Arabidopsis thaliana* seedlings [19]. The seedlings were grown in liquid medium and in a 48-well MTP with a simulated day-night cycle. The cell suspension cultures were treated with the priming-active compounds SA and methyl 1-(3,4-dihydroxyphenyl)2-oxocyclopentane-1-carboxylate (Tyr020). Later they were challenged with flg22, a 22-amino acid peptide. The respiration activity was measured with a modified µRAMOS technique [20] to enable OTR measurement in each single well of the 48-well MTP to draw conclusions about possible priming activity.

The here presented study aimed at developing a high-throughput online screening system to identify priming compounds with plant cell suspension cultures. To do so, the oxygen consumption of parsley suspension cells was measured by monitoring the dissolved oxygen tension (DOT) in each well of a MTP. Monitoring of the DOT was performed using a BioLector device and optical fluorescence spots [21, 22]. The OTR can be calculated from the DOT, if the volumetric mass transfer coefficient (k_L_a) is known and constant [23]. The suitability of this method for the identification of priming compounds has been verified in this work, using the above-described system with SA as a known priming-active compound and the elicitor Pep13 [11, 13]. With this high-throughput method for online measuring the DOT in MTPs, it is possible to identify potential priming compounds. In addition to the OTR measurements, the furanocoumarins were fluorometrically analyzed. The secretion of furanocoumarins can online be monitored via fluorescence measurements on the BioLector platform. Besides furanocoumarins, SA can also be monitored fluorometrically [17]. The measurement of the respiration activity and the fluorescence of SA and furanocoumarins in MTPs were used to monitor the response of parsley cell cultures to treatment with SA and Pep13. With the combination of fluorescence and DOT measurement in MTPs, a noninvasive online high-throughput screening system for priming compounds was developed. In addition, different concentrations and times of treatment with supposed priming compounds can be analyzed.

## Results and discussion

### Establishing parsley cell cultivation in MTPs

For higher throughput at the identification of putative priming compounds, parsley cell culture aliquots were transferred from shake flasks to MTPs. To allow for the comparison with data of previous studies [17] and the scalability between the different cultivation systems, the conditions of cultivation in MTPs should be as similar as possible to those in shake flasks. Shaking frequency was used as the parameter for down scaling. When determining the suited shaking frequency for cell cultivation, three factors need to be considered. First, the shaking frequency needs to be high enough to obtaining a homogeneously suspended cell culture [24, 25]. Therefore, the critical shaking frequency (n_crit_) should be calculated [26]. This parameter is derived from the balance of the centrifugal force and the interfacial tension of the liquid and the walls of the wells. Below the n_crit,_ the centrifugal force is insufficient to overcoming the interfacial tension. This then results in a horizontal liquid surface with heterogeneously mixed fluid. Above the n_crit_, the fluid slides up the wall of the well thus increasing mixing and the mass transfer area. Second, an oxygen limitation must be avoided, to ensure oxygen-unlimited cultivation conditions like those in shake flasks [27–29]. Third, the resolution of the DOT signal needs to be high enough to enable the calculation of OTR values from DOT measurement. The resolution of the DOT signal increases with low, but by no way limiting oxygen content in the liquid at maximum breathing activity. As parsley cell cultures have a generally low oxygen uptake rate [17], the shaking frequency must be sufficiently low to allow for a reasonable resolution of the DOT signal. The three requirements described above are partially conflicting as increasing the shaking frequency needed for sufficient mixing and oxygen-unlimited conditions also increases the DOT resulting in lower DOT signal resolutions.

### Determining a suitable shaking frequency

**Fig. 1:**
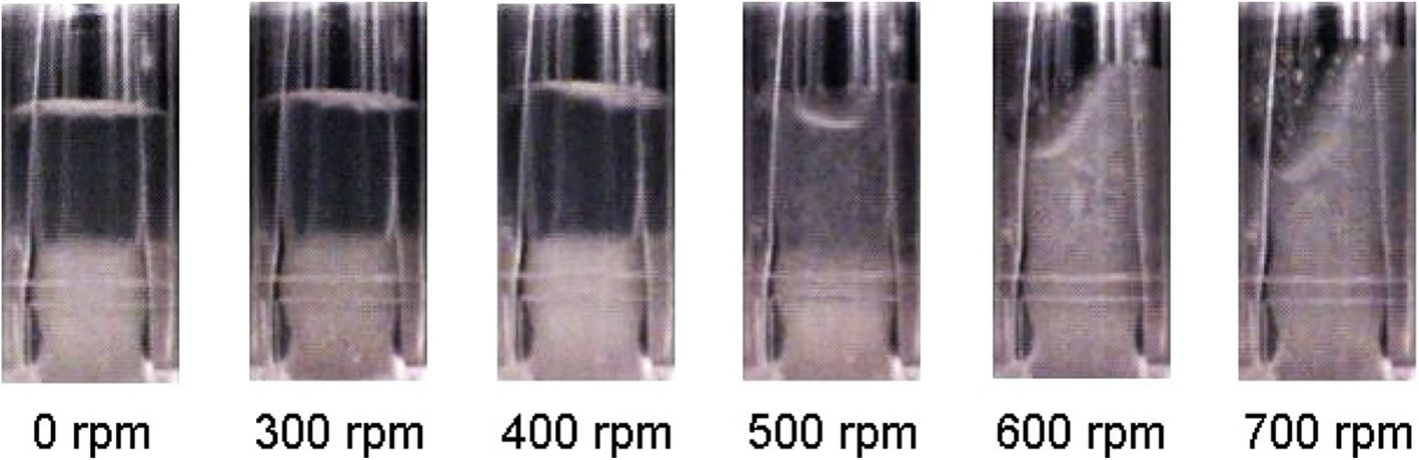
Suspension behavior of a parsley cell culture in a 48-deep-round-well MTP at different shaking frequency. Photos were taken with a CCD camera installed on the shaker platform. An aliquot of a parsley cell culture in a shake flask was transferred, after 120 h of cultivation, to the well of a MTP. Shaking parameters: 48-deep-round-well MTP, shaking diameter d_0_ = 3 mm, filling volume V_L_ = 2 mL, surface tension σ = 0.068 N/m, well diameter D_w_ = 12.4 mm, liquid density σ_L_ = 1.0 kg/L. The contrast and brightness of the photo were edited to distinguish better between the phases with cells in suspension and the unmixed cell broth

Figure 1 displays the suspension behavior of parsley cell cultures in a 48-deep-round-well MTP at varying shaking frequency between 0 and 700 rpm.

From 0 to 400 rpm, the parsley cells are not suspended at all. They rather accumulate at the bottom of well. At 500 rpm, the boundary layer is blurred, and the cells are partially suspended (Fig. 1). At 600 rpm and more, the cells are extensively dispersed in the cultivation liquid. The liquid slides up the wall of well with crescent shape. Hence, shaking at 600 rpm and more seems to be needed for the cultivation of parsley culture cells in the MTPs. However, according to calculations with Eq. 1 (see, “Materials and Methods”), the theoretical n_crit_ for this experimental setup is 188 rpm [26]. One reason for the inconsistency between theoretical and practically determined critical shaking frequency might be the formation of parsley cell aggregates [24, 25]. A similar effect was observed when mixing water with cellulose fibers [28]. The authors reported that cellulose particles also were suspended at a shaking frequency that was far above the theoretical n_crit_ [30]. Thus, one can assume that the presence of cell aggregates could increase the shaking frequency needed for homogeneous dispersion of cells. Together, we found that a shaking frequency of 600 rpm and more is needed to achieve sufficient homogenization of the parsley cells in suspension in MTPs.

### Volumetric mass transfer coefficient and OTR calculations

To assess whether the parsley cell cultures in MTPs are limited in oxygen, the maximum oxygen transfer capacity (OTR_max_) given in a fixed set of cultivation conditions is required. According to the data of m2p-labs (now Beckman Coulter) and the literature [28, 31], the OTR_max_ is 6.1 mmol/L/h at the chosen cultivation conditions (48-deep-round-well MTPs, V_L_ = 2 mL, n = 600 rpm, d_0_ = 3 mm and 25 °C). This value was determined with a 0.5 M sulfite oxidation system [28] by m2p-labs (now Beckman Coulter) and is shown in the technical data sheet for MTP-R48 plates. The oxygen solubility of the sulfite system differs from the modified Gamborg’s B5 medium used for cultivating the parsley cells. The oxygen solubility of the sulfite system is 0.84 mmol/L/bar, whereas the oxygen solubility of the modified Gamborg’s B5 medium is 1.22 mmol/L/bar (See Materials and methods: Determination of the DOT and OTR) [32–34]. The OTR_max_ increases with increasing oxygen solubility (Eq. 4, see Materials and Methods). Consequently, the OTR_max_ for the Gamborg’s B5 medium is expected to be above 6.1 mmol/L/h. Parsley cell cultures in previous shake flask experiments reached an OTR of 3—4 mmol/L/h [17]. Consequently, no oxygen limitation seems to occur in MTPs at the chosen cultivation conditions.

**Fig. 2:**
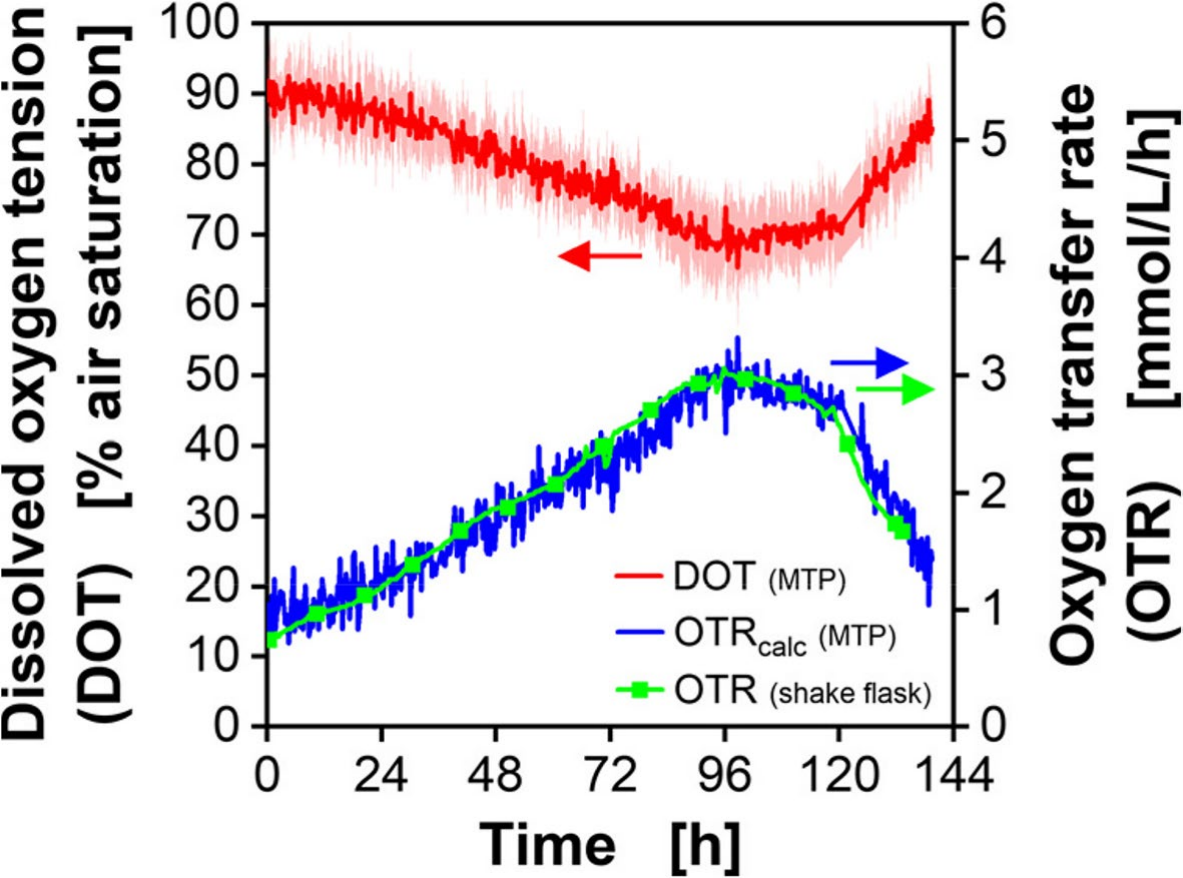
Respiration activity of parsley cell cultures, determined in a shake flask and a 48-deep-round-well MTP. Air-supplied (p_O2_ = 0.21 bar) parsley cell cultures were simultaneously cultivated in 250-mL shake flasks using RAMOS device (green curve) and in 48-deep-round-well MTP using a custom made BioLector device from m2p-labs (now Beckman Coulter). Mean values for the DOT (red) were calculated from 10 wells. The standard deviation (SD) is indicated by blue and red shadows. OTR_calc_ in the MTP is calculated from the measured DOT according to Eq. 3. Parameters: k_L_a = 37 1/h, L_O2_ = 1.22 mmol/L/bar, partial pressure of oxygen in air p_O2_ = 0.21 bar. Shake flasks cultivation conditions: V_L_ = 50 mL, *n* = 180 rpm, d_0_ = 50 mm and 25 °C in modified Gamborg’s B5 medium. For clarity, only every twentieth measuring point of the OTR RAMOS curve is marked as a square symbol. MTP cultivation conditions: V_L_ = 2 mL, *n* = 600 rpm, d_0_ = 3 mm and 25 °C in modified Gamborg’s B5 medium. PreSens sensor spots were used for measuring the DOT in MTPs

For comparison of results with shake flasks and MTPs, the OTR in the MTP was calculated from the measured DOT. To do so, the volumetric mass transfer coefficient (k_L_a) had to be determined (see, Materials and Methods). The k_L_a depends on the well geometry, plate material and the cultivation conditions, which include the shaking frequency, filling volume and shaking diameter [35]. It is possible to determine the k_L_a in MTPs by simultaneous measurement of the OTR in shake flasks and the DOT in MTPs at oxygen-unlimited condition. This method has been published for microbial cultures [23, 36]. Equation 3 shows the relationship between OTR and DOT and enables the estimation of the k_L_a value in the MTP to be 37 1/h (see, Materials and Methods). When knowing the k_L_a value in the MTP, the OTR (OTR_calc_) in the MTP can be concluded from the DOT (see, Eq. 3). Figure 2 shows the DOT and OTR of parsley cells cultivated in MTPs (red and blue curves) and the OTR obtained in shake flasks (green curve) cultivated simultaneously.

The DOT in the MTPs decreased from 90% to almost 70% at 96 h and increased afterwards. The OTR during shake flask cultivation shows an inverse courese with a maximum of 3 mmol/L/h after 96 h before it decreased again. To compare the calculated data with the measured ones, the OTR_calc_ that we calculated from the DOT values of the MTP is shown in blue in Fig. 2. The similarity in OTR progression in shake flasks and MTPs discloses similar culture behavior and a seemingly correct estimation of the k_L_a value in the MTP. Consequently, the scale-down of parsley cell cultures from shake flask to MTPs has been successful. The selected cultivation conditions (V_L_ = 2 mL, *n* = 600 rpm, d_0_ = 3 mm) are suitable for cultivating parsley cells in 48-well MTPs. In addition, we demonstrated that the signal of the DOT optode in chosen cultivation conditions was suited to follow the culture progression with sufficient resolution.

### Analyzing priming compounds in MTPs

**Fig. 3:**
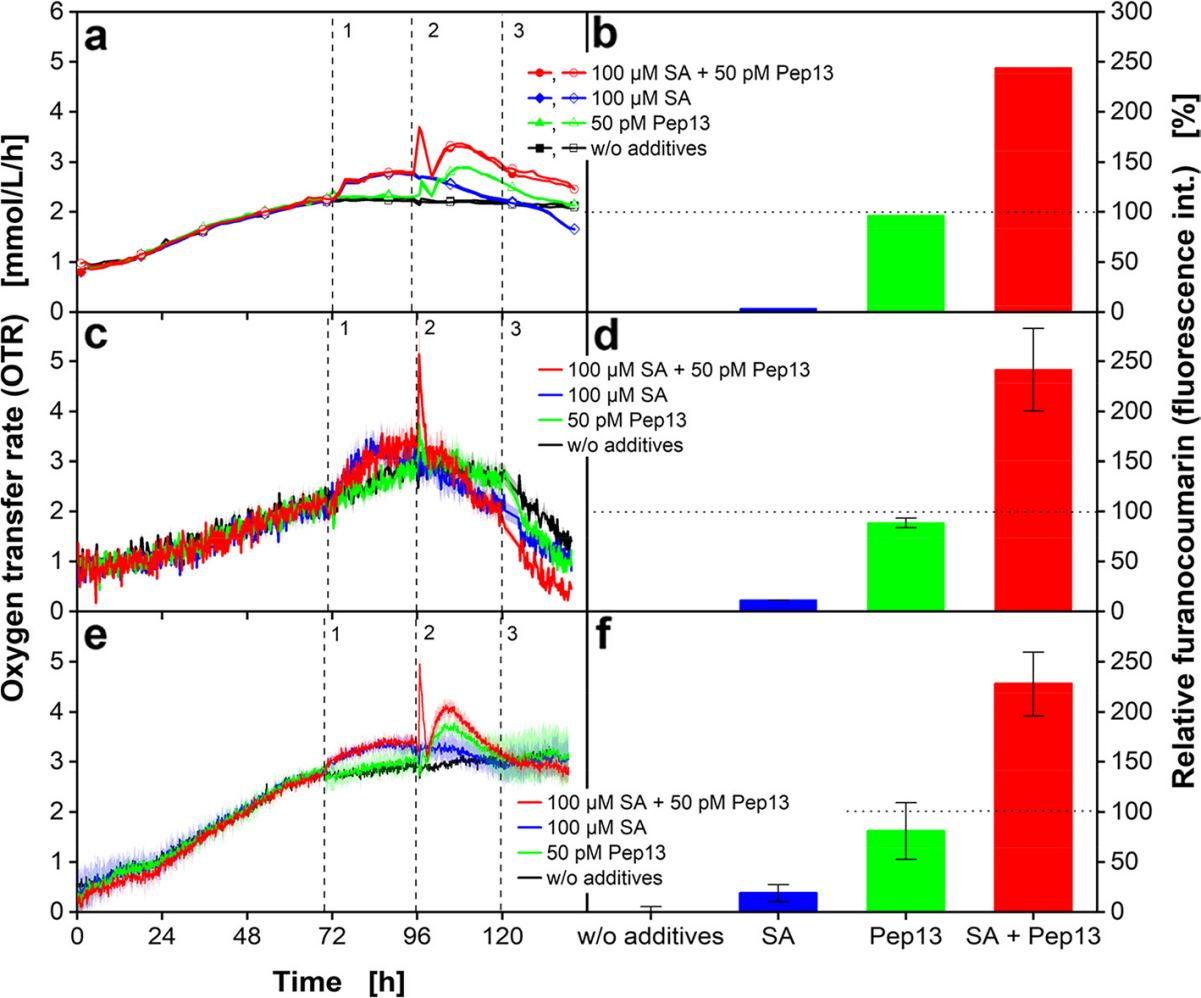
OTR of parsley cell cultures in shake flasks and MTPs with two different sensor systems. Measurements from shake flasks (**a**) and from MTPs (**c**, **e**) are shown. Corresponding relative furanocoumarin fluorescence intensities at 120 h of cultivation in cultures primed with SA and elicited with Pep13 (**b**, **d**, **f**). Parsley cell cultures were cultivated in 250 mL shake flasks using the RAMOS device (**a**, data from Schilling et al. 2015), in 48-deep-round-well MTPs with PreSens sensor spots using a custom-made industrial BioLector device (**c**) and with PyroScience sensor spots using an in-house built BioLector (**e**) for measuring the DOT. Dashed vertical lines (**a**, **c**, **e**) indicate the addition of 100 µM SA (1), 50 pM Pep13 (2) and offline fluorescence measurement of furanocoumarins (3). Shake flask cultivation conditions: 250 mL RAMOS flasks, V_L_ = 50 mL, *n* = 180 rpm, d_0_ = 50 mm, 25 °C in modified Gamborg’s B5 medium. For the OTR (**a**) two replicates are shown. Every tenth measuring point of the OTR curve is marked as symbol (**a**). MTP cultivation conditions: V_L_ = 2 ml, *n* = 600 rpm, d_0_ = 3 mm and 25 °C in modified Gamborg’s B5 medium. Mean values were calculated from 10 wells (**c**) and from three wells (**e**), respectively. Data were normalized (see Material and Methods) and the SD is indicated by colored shadows and (**c**, **e**). The OTR in (**c, e**) is calculated from the DOT according to Eq. 3. Parameters for MTP fluorescence measurements in the in-house built BioLector: *n* = 600 rpm, d_0_ = 3 mm and 25 °C. Relative furanocoumarin fluorescence (**b**, **d**, **f**) was calculated according to Eq. 6. Dotted horizontal lines represent the 100% threshold. Since the red curves in (**c**, **e**) cover partly the green curves, the graphs are shown without the red curves in the Additional file 1c and g

After finding out suitable conditions of parsley cell cultivation, we investigated whether the online data obtained from cultivation in MTPs would allow for drawing conclusions as to whether a chemical compound in question would possibly be a defense priming-activating compound in plants. As shown before [17], the treatment of parsley cell cultures with priming-activating compounds and subsequent elicitation lead to more robust OTR. Because the measurements in the MTPs actually do not detect the OTR but rather the DOT, two different sensor spots were tested for their suitability of detecting the DOT. One of the sensors was obtained from PreSens (m2p-labs (now Beckman Coulter GmbH), Baesweiler, Germany) and the second one from PyroScience (OXSP5, PyroScience GmbH, Aachen, Germany). For evaluation, the DOT measurements of the two sensor-spots systems were compared to the OTR curves obtained with shake-flask cultivations of parsley cells. The optical sensor spot from PreSens measures the DOT at an excitation wavelength (λ_ex_) of 520 nm and an emission wavelength (λ_em_) of 600 nm. The sensor spot of PyroScience rather uses a λ_ex_ of 610 nm and λ_em_ of 760 nm. All experiments were done in oxygen-unlimited condition. The OTR_max_ in the RAMOS-using experiment (Fig. 3a) was calculated as 7.7 mmol/L/h with a k_L_a of 43 h^−1^ [37].

Figure 3 shows the online-recorded OTR and fluorescence of SA treated parsley cell cultures in shake flasks (Fig. 3a, b) and MTPs (Fig. 3c, d, e, f).

Figure 3a and b show data from Schilling et al. (2015) as a reference, to evaluate the novel results obtained with cell cultures in MTPs as shown in Fig. 3c, d, e, and f. The OTR values in Fig. 3c and e were calculated with Eq. 3 and are based on the measured DOT. The raw DOT data with their inverse course is shown in Additional file 1a and e. Since the course of the red curve in Fig. 3c and e matches those of the green and blue curves, the same graph is plotted also with omitted red curve in Additional file 1c and g. For Fig. 3c, the DOT was measured with PreSens sensor spots, while the DOT for Fig. 3e was determined with PyroScience spots. In Fig. 3b, d and f, the relative furanocoumarin fluorescence after 120 h was calculated using Eq. 6. The fluorescence measurements are used as reference to assessing whether suspension cells were in an alert state of defense in the MTP. Relative furanocoumarin fluorescence intensities over 100% for cultures subsequently treated with priming compound first and elicitor second indicate a successful priming experiment. That is because primed cells secreted much more furanocoumarins than non-primed ones [11, 17]. For reference, the furanocoumarin fluorescence of naïve cell cultures (e.g. with no addition of SA and Pep13; Fig. 3a, c, e, black curve) and cultures treated with either SA (Fig. 3a, c, e, blue curve) or Pep13 (Fig. 3a, c, e, green curve) are shown.

In experiments with both shake flask and MTPs, treatment with SA followed by elicitation with Pep13 lead to about 2.5-fold enhancement of furanocoumarin fluorescence (Fig. 3b, d, f). As the relative furanocoumarin fluorescence in Fig. 3b, d and f are similar, the treatment of parsley cell cultures with SA and Pep13 in MTPs and shake flasks seems to mobilize similar physiological responses, independent of the cultivation container.

Comparing the OTR in Fig. 3a, c and e, the addition of SA at 72 h post cultivation lead to an OTR increase (blue curve). In the cultures experiencing Pep13 addition at 96 h, the OTR shows a short peak and a prolonged rise in the OTR (green curve). The peak and following increase of the breathing activity after Pep13 addition is much higher in the cultures that were treated with SA before Pep13 elicitation (red curve).

The calculated OTR values obtained in MTP using PreSens spots (Fig. 3c) have a higher scatter than the OTR curves shown in Fig. 3a (shake flask). This is most likely caused by a technical problem but was not analyzed in detail as the signals from PyroScience spots show good resolution. The different standard deviations and resolutions of the OTR curves in Fig. 3c and e, as measured with the different sensor spots, are also remarkable. The DOT signals from the PreSens spots (Fig. 3c) are noisy, which prevents a distinct differentiation of the primed and unprimed parsley cell cultures.

The calculated OTR curves from the MTP with PyroScience spots (Fig. 3e) shows better resolution, less background noise, and lower standard deviations, compared to the calculated OTR curves from the MTP with PreSens spots (Fig. 3c). The data generated with the PyroScience spots show the same qualitative characteristics as the OTRs in Fig. 3a, obtained in shake flasks. The PyroScience sensor spots (Fig. 3e) deliver a signal with lower scattering than the sensor spots from PreSens (Fig. 3c), although the depicted mean values were calculated from 3 versus 10 parallel wells respectively. The OTRs calculated from the DOT using PyroScience spots (Fig. 3e) reach slightly higher overall values, compared to shake flasks (Fig. 3a). The reference cultures without any treatment in Fig. 3a reached an OTR of approximately 2.2 mmol/L/h, whereas the reference cultures without any treatment in Fig. 3e reached around 3.0 mmol/L/h. This difference can be attributed to independent experimental setup and inoculums. The significance in DOT values was validated by a 5% significance level students’ t-test and is shown in Additional file 1d and h. It was demonstrated that differences in the OTR were successfully determined from DOT measurements in MTPs, using sensor spots from PyroScience. The experiments in MTPs led to similar results as previous experiments with parsley cell cultures in shake flasks [17]. The low throughput in shake flasks is a major drawback for rapid and easy testing of priming-active compounds. With one RAMOS device, the simultaneous measurement of only eight flasks is possible. If for all experimental variants duplicates are used, two flasks are used for negative controls without treatment. Additionally, two flasks are used for negative controls with Pep13. This leaves four flasks to measure a single new test substance. Two of the flasks are used for negative controls of the test substance and the other two flasks for the treatment with test substance and subsequent Pep13 treatment. Thus, one single test substance can be analyzed in one RAMOS experiment. In a 48-well MTP, two wells are also used for the negative control without treatment and two wells for the negative controls of Pep13. This leaves 44 wells for the investigation of new test substances including their respective negative controls. Four wells per new test substance are required, two wells for the negative controls with the test substance and two wells for the cultivations treated with test substance and Pep13. Therefore, 11 new substances can be tested per 48-well MTP, which translates into an 11 times higher throughput of 48-well MTPs in relation to RAMOS experiments with shake flasks. This increased throughput will simplify the screening for priming-active compounds.

### Reduced oxygen supply

Although the resolution of the signals from the MTP experiment with PyroScience spots is already significant in the decisive cultivation period after Pep13 addition (Additional file 1h), a further improvement of the signal quality is desirable for the application with other putative but possible weaker priming-activating compounds. This would increase the detection of priming-active compounds with even lower effect on defense priming than SA. The overall signal quality should increase, if lower DOT values are reached during the cultivation. A better resolution at lower DOT values is obtained, as the relationship between the DOT and the fluorescence signal intensity is not linear. Because of the Stern–Volmer relationship, the sensor spot displays a low signal resolution at high oxygen concentrations. Therefore, experiments at reduced DOT levels were performed. The oxygen content of the supply air was adjusted to p_O2_ = 15% by mixing air and nitrogen. The DOT values and calculated OTR curves are shown in Additional file 2. Compared to the OTR from shake flask cultures aerated with air (p_O2_ = 21%) (Additional file 2b, black curve), the calculated OTR from MTPs shows clearly lower overall values. The DOT at a p_O2_ = 15% (Additional file 2a) is only slightly reduced when compared to the experiment with air aeration (p_O2_ = 21%) (Additional file 1e). The lower OTR indicates an impaired oxygen uptake. A possible reason for this observation may lie in sensitivity against oxygen level changes of the parsley cell cultures. The plant cells might sense the reduced oxygen content in the gas phase and change their metabolism. The changed metabolism may lead to a reduced breathing activity. This kind of response is documented for whole plants and should be considered in further experiments [38–40]. Since no signal improvement could be achieved at reduced oxygen concentration in the gas supply, this approach was not pursued further in this study.

Another technique of measuring the OTR in MTPs is the µRAMOS approach [20, 41, 42]. µRAMOS enables OTR measurements in any individual well of a 48-well MTP applying oxygen sensitive fluorescence sensor spots. The µRAMOS technique is also suitable to investigate defense priming of parsley suspension cell cultures. In contrast to the DOT measurements via the fluorescence sensor spots with a spectrofluorometer, no mathematical conversions of DOT values to OTR are necessary. µRAMOS measurements showed good resolution in measurements with parsley suspension cell cultures [20]. However, measuring the DOT using the fluorescence sensor spots provides additional information. It reports whether the cultures are well supplied with oxygen and whether they are approaching oxygen limitation. This is important information, especially for work with plant suspension cell cultures, which seem to be very prone to varying oxygen levels (Additional file 2). In addition, with a spectrometer for DOT measurements, it is possible to online monitor additional fluorescence signals (see, the next paragraph).

### Online fluorescence measurement in MTPs

As shown above, defense priming can be analyzed in MTPs by measuring the DOT signal. In addition, the cultivation of cells in the BioLector device enables the measurement of fluorescence signals, including those of SA at λ_ex_ = 295 nm and λ_em_ = 405 nm and secreted furanocoumarins of the parsley cells upon priming, at λ_ex_ = 335 nm and λ_em_ = 398 nm [43]. The concentration of SA and furanocoumarins can be measured online, simultaneously to the breathing activity, providing additional insights on the culture behavior.

**Fig. 4:**
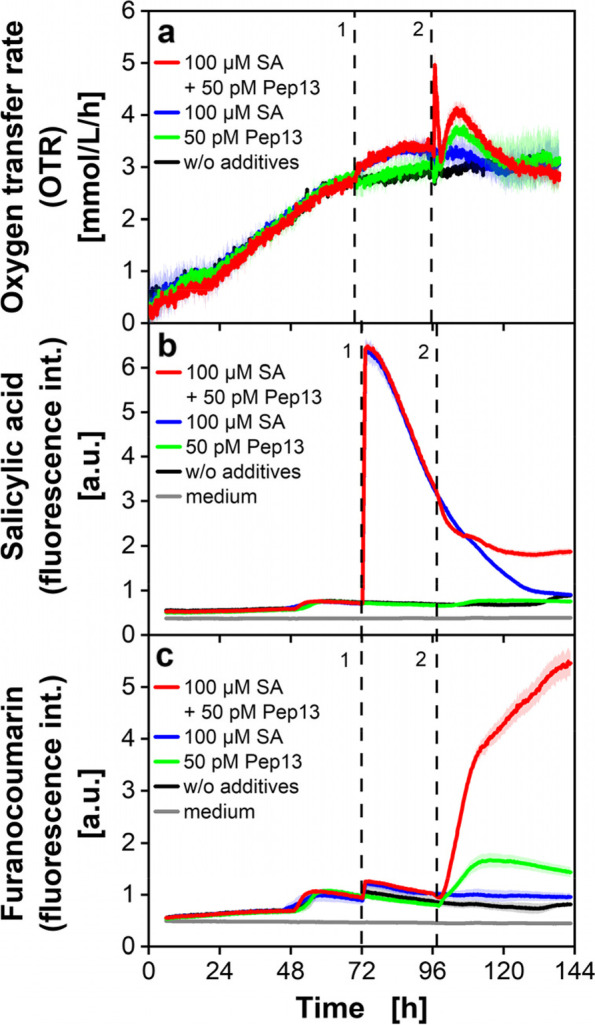
OTR and online determination of furanocoumarin fluorescence of parsley cell cultures in an MTP. Parsley cells were cultivated in a 48-deep-round-well MTP with PyroScience sensor spots, aerated with air (p_O2_ = 0.21 bar) in an in-house built BioLector devise. The OTR was calculated from the DOT according to Eq. 3 and plotted in Figure (**a**). In a different experiment, SA (**b**) and furanocoumarin fluorescence (**c**) were measured by an in-house built BioLector in a 48-deep-round-well MTP. Wavelength combination for SA fluorescence measurement: λ_ex_ = 295 nm and λ_em_ = 405 nm (**b**) and furanocoumarin fluorescence: λ_ex_ = 335 nm and λ_em_ = 398 nm (**c**). Mean values were calculated from 3 wells in **a** and from 5 wells in **b** and **c**. Colored shadows indicate SD. Dashed vertical lines indicate the times of addition of SA (1) and/or Pep13 (2). Cultivation conditions: V_L_ = 2 mL, *n* = 600 rpm, d_0_ = 3 mm and 25 °C in modified Gamborg’s B5 medium

In Fig. 4a, the course of the calculated OTR from Fig. 3e is depicted and compared to the online fluorescence signals of SA (Fig. 4b) and furanocoumarins (Fig. 4c) during cultivation.

In general, the reproducibility of the fluorescence data was excellent. The standard deviations as calculated from 5 wells are depicted as colored shadows and are very small. In most cases the standard deviation is hardly visible. The OTR (Fig. 4a) increases after SA addition at 72 h (red and blue curve) and again after the Pep13 addition at 96 h (red and green curve). The SA fluorescence intensity (Fig. 4b all curves) increases slightly for the first time after 48 h, although no addition took place at this time. A change in metabolism could cause this increase, due to substrate limitation in the medium of the cell cultures [44, 45]. After addition of SA at 72 h (Fig. 4b, red and blue curve), the SA fluorescence increases rapidly [43]. Afterwards, the fluorescence signal in those cultures within 58 h dropped to their initial value (blue curve). The decrease in SA fluorescence might be due to conversion of SA to its presumed storage form SA-glucoside, as reported for parsley, soybean, and tobacco [46–50]. SA fluorescence intensity is influenced by Pep13 addition, resulting in a lower decrease after 108 h (Fig. 4b, red curve). The elicitor Pep13 triggers defense mechanisms of parsley cell cultures [12]. The slower decrease of the SA fluorescence in cultures treated with SA and Pep13 might point towards a priming effect, as SA remains longer in primed cells.

In Fig. 4c, the signal of secreted furanocoumarins slightly increases, if SA was added to the cells after 72 h (red and blue curve). This increase might be due to the overlapping spectra of SA and furanocoumarins [43]. With previous priming, the Pep13 addition leads to a substantial increase in the furanocoumarin fluorescence (Fig. 4c, red curve). But also, without priming by SA, the furanocoumarin fluorescence clearly increases (Fig. 4c, green curve), as it is triggered by the addition of Pep13. Compared with the OTR, SA fluorescence and furanocoumarin fluorescence data match very well and confirm the success of the cell pretreatment. Another recently published article presents a screening method for priming-active compounds in 24-well MTPs [14, 51]. This method relies on the detection of furanocoumarins with UV light. Parsley cell cultures are first incubated in a shake flask and are then distributed in 24-well MTPs. In the MTP the cell cultures are treated with defense priming compounds and an elicitor, to trigger the plants immune system. The immune response is validated by measuring the furanocoumarin fluorescence by transferring the supernatant to an MTP in a microplate reader for fluorescence measurement. Thereby, a straightforward and easy to apply screening system was demonstrated. However, this method is based on an offline endpoint measurement. The study presented in this work shows that the fluorescence properties of furanocoumarins are also detectable online in 48-well MTPs. This increases the throughput even more and allows a more detailed online analysis of the priming procedure.

### Varying SA concentrations for cell pretreatment

The possibility to online monitor the fluorescence signals of SA and furanocoumarins in MTPs enables a detailed investigation of the effect on defense priming of different priming compounds, concentrations, and combinations for priming compound and elicitor addition times. In the following section, different concentrations of the model priming compound SA are analyzed.

**Fig. 5:**
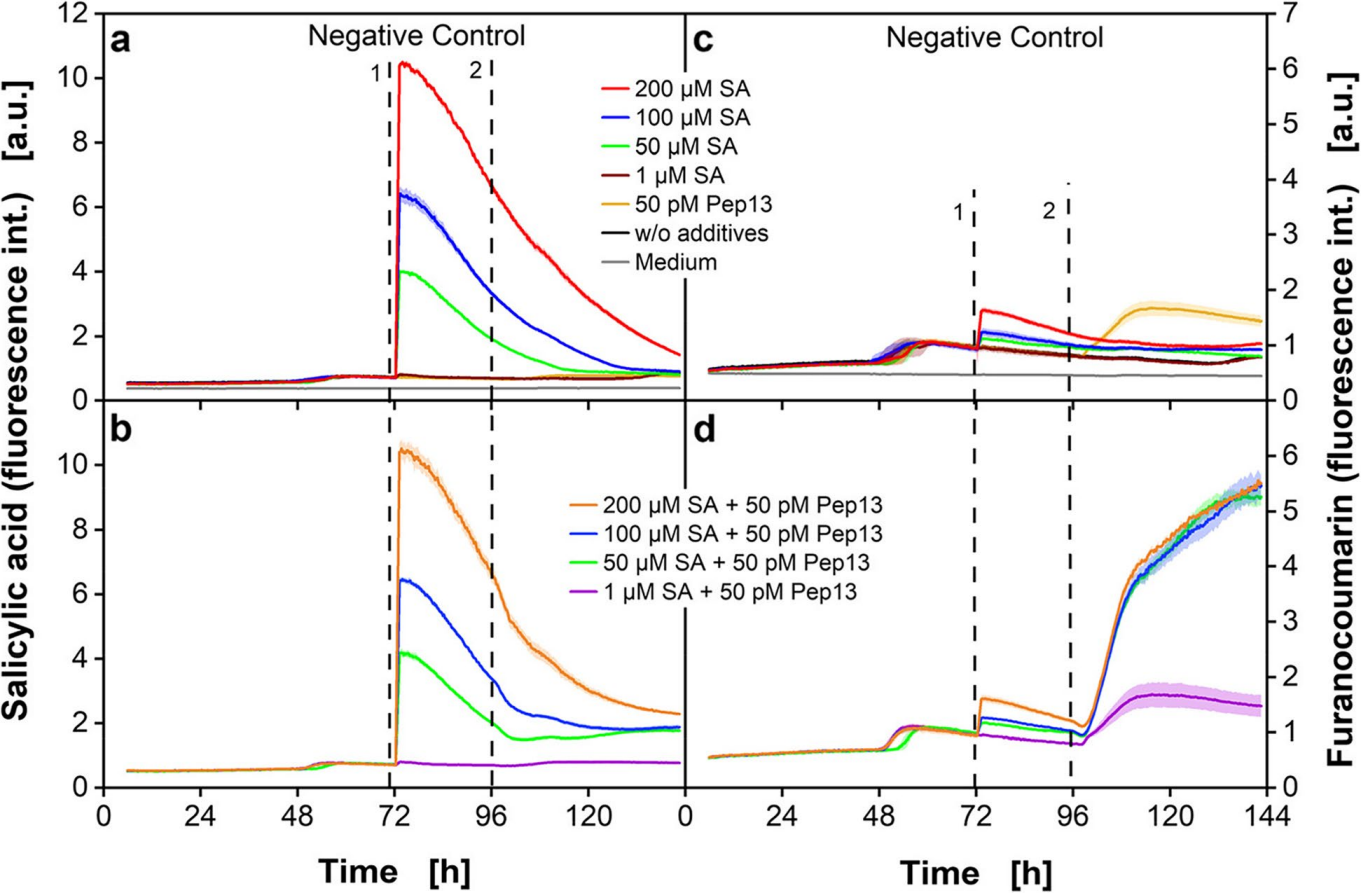
Online fluorescence intensities upon treatment with various SA concentrations in a MTP. Parsley cells were cultivated in 48-deep-round-well MTPs aerated with air (p_O2_ = 0.21 bar) in an in-house built BioLector. SA fluorescence was measured at λ_ex_ = 295 nm and λ_em_ = 405 nm (**a**, **b**) whereas furanocoumarin fluorescence was determined at λ_ex_ = 335 nm and λ_em_ = 398 nm (**c**, **d**). Mean values were calculated from three wells. SD is indicated as colored shadows. Dashed vertical lines indicate the addition of SA (1) and 50 pM Pep13 (2). Cultivation conditions: V_L_ = 2 mL, *n* = 800 rpm, d_0_ = 3 mm and 25 °C in modified Gamborg’s B5 medium

Figure 5 shows the fluorescence signals for cell cultures treated with SA concentrations ranging from 1 µM to 200 µM at constant Pep13 concentration (50 pM).

Addition times of 72 h for SA and 96 h for Pep13 are used. In Fig. 5a and c, the negative controls with either SA or Pep13 addition are depicted. The SA fluorescence signal (Fig. 5a) shows a dose-dependent increasing fluorescence intensity with increasing SA concentration. After SA addition at 72 h (Fig. 5a, dashed black line marked with 1), the fluorescence signal decreases for 48 h – 72 h. Only the SA concentration of 1 µM (Fig. 5a, brown curve) is not distinguishable from the culture without SA addition (Fig. 5a, black curve) and neither increases nor decreases.

In Fig. 5c, the furanocoumarin signal shows a slight increase after SA addition at 72 h (Fig. 5c, dashed black line). The increase is more substantial with higher SA concentrations. As mentioned before, this might be caused by overlapping fluorescence spectra of SA and furanocoumarins [43]. The treatment with Pep13 after 96 h (Fig. 5c, yellow curve) also increases the furanocoumarin fluorescence, as it triggers the defense mechanisms of the plant cells to a limited extend. The fluorescence signals of the cultures treated with SA after 72 h and Pep13 after 96 h are shown in Fig. 5b and d. In Fig. 5b a dose-dependent increase of SA fluorescence, except for 1 µM SA is demonstrated. For subsequent addition of 50 µM SA and Pep13 (Fig. 5b, green curve), the SA fluorescence signal even increases slowly after 100 h of cultivation. For this increase a combination of a slower degradation and an overlap with the increasing furanocoumarin fluorescence signal may be responsible [43]. In Fig. 5d, the furanocoumarin fluorescence signals do not show dependence of dose. The addition of 1 µM SA (Fig. 5d, violet curve) seems to be a too low concentration for measuring defense priming with the here used fluorescence technique. With 50 µM (Fig. 5d, green curve), 100 µM (Fig. 5d, blue curve), and 200 µM (Fig. 5d, yellow curve) SA priming results in the same furanocoumarin production over time. It is possible, though, that dose-dependent differences may become visible at a later stage of cultivation. The results show that it is generally possible to use lower SA concentrations for effective priming of parsley cell cultures, as the furanocoumarin synthesis for the cultures primed with 50 µM, 100 µM or 200 µM SA was identical until 144 h cultivation time. In future, concentrations of SA between 1 µM and 50 µM could be investigated to identify the lowest concentration necessary for successful priming. Our results disclose the need of continuous online monitoring and the relevance of fluorescence-based analyzes in MTPs to thoroughly understand plant defense priming.

### Varying the times of combined SA/Pep13 addition.

**Fig. 6:**
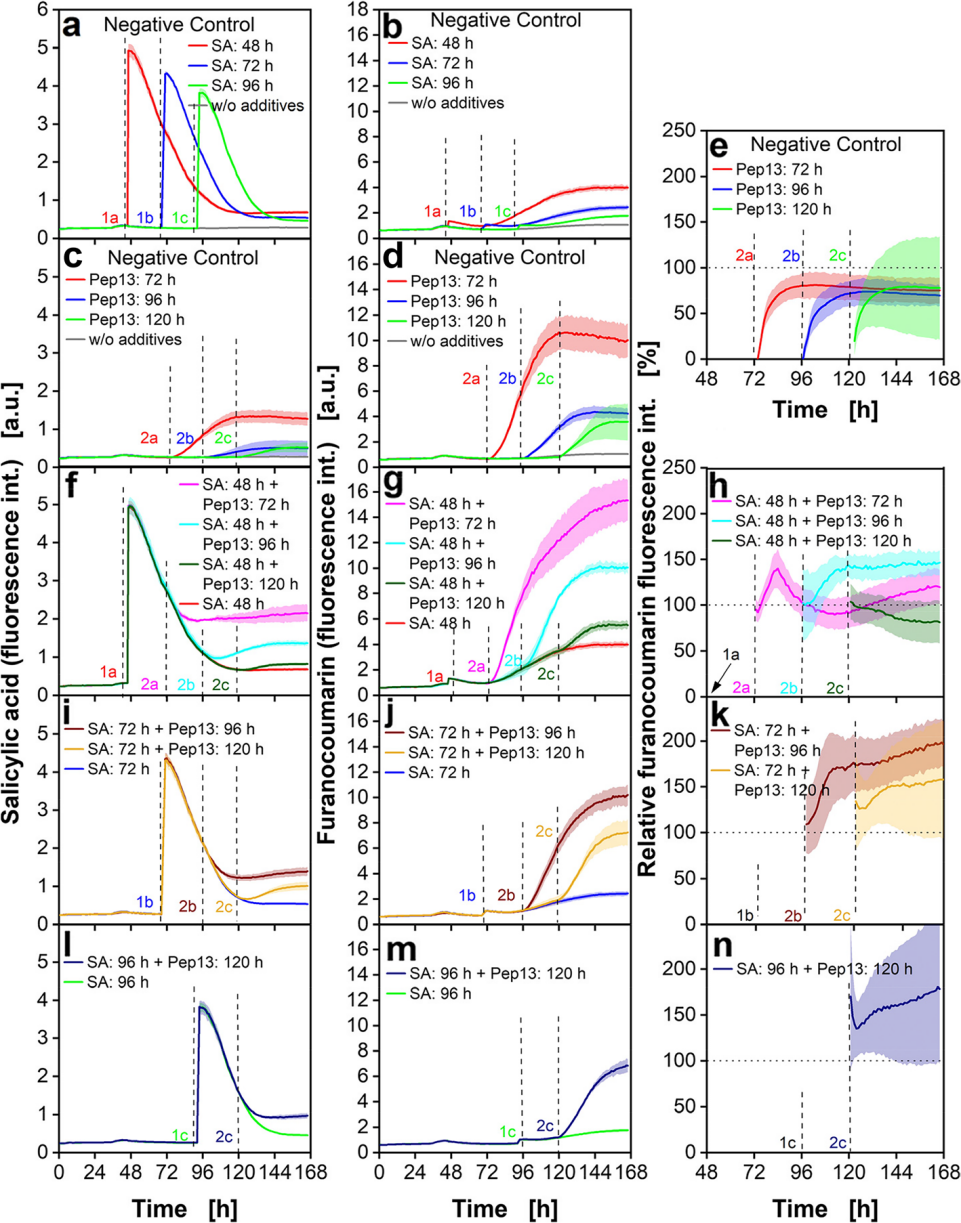
Online fluorescence of parsley cell cultures treated with SA and Pep13 at various cultivation times. Parsley cells were cultivated in 48-deep-round-well MTPs aerated with air (p_O2_ = 0.21 bar) in an in-house built BioLector. Measurement of SA fluorescence at λ_ex_ = 295 nm and λ_em_ = 405 nm (**a**, **c**, **f**, **i**, **l**) and of furanocoumarin fluorescence at λ_ex_ = 335 nm and λ_em_ = 398 nm (**b**, **d**, **g**, **j**, **m**). The negative controls were treated with either 100 µM SA at 48 h, 72 h, and 96 h (**a** and **b**) or with 50 pM Pep13 at 72 h, 96 h and 120 h (**c** and **d**). The calculated relative furanocoumarin fluorescence intensity is shown in **e**, **h**, **k** and **n** and was calculated according to Eq. 6. Dotted horizontal lines in Fig. 6 **e**, **h**, **k** and **n** represent the 100% threshold value which is the sum of the corrected fluorescence intensities of the cultures treated exclusively with SA or Pep13 (Materials and Methods: Fluorescence measurements of SA and furanocoumarins). Relative furanocoumarin fluorescence intensities over 100% for cultures treated with both, priming compounds and elicitor, indicate a successful priming. Mean values were calculated from three wells. SD is indicated by colored shadows. The SD for the relative furanocoumarin fluorescence intensity (**e**, **h**, **k**, **n**) was calculated by error propagation as described in “Materials and Methods”. Dashed vertical lines indicate the addition of 100 µM SA (1a: ~ 48 h, 1b: ~ 72 h, 1c: ~ 96 h) and 50 pM Pep13 (2a: ~ 72 h, 2b: ~ 96 h, 2c: ~ 120 h) at varying time points. Cultivation conditions: V_L_ = 2 mL, *n* = 800 rpm, d_0_ = 3 mm and 25 °C in modified Gamborg’s B5 medium

In the above experiments, SA was applied to parsley cell cultures at 72 h and Pep13 at 96 h of cell cultivation. Figure 6 shows the fluorescence signal of SA and furanocoumarins after combined addition of SA and Pep13 at varying times.

The columns displayed in Fig. 6 give the values of SA fluorescence, furanocoumarin fluorescence, and relative furanocoumarin fluorescence. The latter allows a direct conclusion as to the time combinations that lead to the strongest defense priming seen in the parsley cell cultures, without the necessity to compare the fluorescence data for any single experiment with the negative controls. In the first two rows of Fig. 6 (a, b, c, d and e), the control treatment of either SA or Pep13 are shown. In rows 3 to 5 of Fig. 6 (f, g, h, i, j, k, l, m, and n), the fluorescence intensities of SA and Pep13-treated cultures are displayed. SA was added at 48 h, 72 h or 96 h. We added Pep13 after 72 h, 96 h or 120 h. Dashed vertical lines indicate the addition time points and are specifically numbered. Rows 3 to 5 show the different addition time points of SA combined with various Pep13 addition time points. All graphs of Fig. 6 indicate that SA and furanocoumarin fluorescence increase to higher levels if SA or Pep13 are added earlier. One explanation for the higher fluorescence signals of younger cultures may be a higher cell density at later cultivation times, which interferes with the fluorescence signal. Another assumption is that with the same concentrations of SA and Pep13 but higher cell densities, the concentration of the priming compound per cell and the resulting furanocoumarin fluorescence are lower. The SA fluorescence signals strongly increase in response to SA, followed by a decrease during several days (Fig. 6a, c, f, i, l). The period of decreasing SA fluorescence is shorter, if SA is added later. These observations are possibly because of faster metabolization of SA by the cells in higher density in aged cultures. Figure 6d depicts that also the Pep13-elicited increase in furanocoumarin fluorescence was faster and higher when cells were elicited at early rather than late times of cultivation. Different treatment times with Pep13 result in the synthesis of different amounts of furanocoumarin (Fig. 6d, g, j, m). A possible explanation for the varying response of the cells after Pep13 treatment is a growth state-dependent reaction of parsley cell cultures [52, 53].

To correctly evaluate the priming effect at different addition times of SA and Pep13, it is helpful to consider the relative furanocoumarin fluorescence intensity. Applying the relative fluorescence, priming at different addition times was compared. Consequently, the relative furanocoumarin fluorescence was calculated by Eq. 6, as described in Material and Methods. The relative furanocoumarin fluorescence is shown in the third column (Fig. 6e, h, k and n). To calculate the relative furanocoumarin fluorescence, the furanocoumarin fluorescence intensity of the negative controls with either SA or Pep13 and the intensity of a SA and Pep13 treated culture are set into relation. The furanocoumarin fluorescence increases after the addition of SA or Pep13. The sum of the negative controls with SA and Pep13 are set to 100%. It is defined that up to this level no priming is existent. Relative furanocoumarin fluorescence above the horizontal dotted line at the 100% threshold indicate increased furanocoumarin production due to previous successful priming. Figure 6e shows only negative controls with either SA or Pep13 treated cultures. Defense priming should not be measureable in the negative controls. The relative furanocoumarin fluorescence should stay below 100% for this negative controls. The relative fluorescence below 100% fits the expectations and shows that no priming effect was measured.

For the addition of SA at 48 h followed by different addition times of Pep13, the relative fluorescence is plotted in Fig. 6h. As can be seen, the relative fluorescence for the addition time of Pep13 of 72 h (Fig. 6h, magenta curve) and 96 h (Fig. 6h, turquoise curve) reaches approximately the same maximum value of 150% and shows successful priming. However, the course of the two curves is different. While for an addition time of Pep13 of 48 h (Fig. 6h, magenta curve) the curve rises at first and reaches a maximum of 150% after 12 h. Afterwards, it decreases and even falls below the 100% limit. In contrast, the curve for the addition time of Pep13 of 96 h (Fig. 6h, turquoise curve) rises to 150% within 20 h after Pep13 addition and remains stable on this plateau. For the addition time of Pep13 of 120 h (Fig. 6h, dark green curve), the relative fluorescence remains below the 100% threshold. This indicates that a time-shift of 72 h between SA addition and Pep13 treatment is too long to reliably detect priming.

The combination of SA addition at 72 h and Pep13 treatment at 96 h (Fig. 6k, dark brown curve) results in the most potent immune response of the experiment, with relative fluorescence of around 200%. This confirms the suitability of the previously used standard test system as described in the literature [10, 11, 13]. The light brown curve in Fig. 6k also demonstrates priming with the combination of SA addition at 72 h and Pep13 treatment at 120 h, however, to a weaker extend, than if Pep13 is added after 96 h.

The graphs in Fig. 6l, m and n (dark blue curve) show the combination of SA addition at 96 h and Pep13 treatment at 120 h. Regardless of the lower SA and furanocoumarin fluorescence values (Fig. 6l and m), compared to earlier addition times, the relative furanocoumarin fluorescence shows a potent immune response upon Pep13 treatment. The relative fluorescence (Fig. 6n) is permanently above 100% after exposure to Pep13 and increases to 180% at the end of the cultivation.

The fluorescence data in Fig. 6 illustrate that the time of addition of SA or Pep13 has a decisive influence on the development of the SA, furanocoumarin and relative furanocoumarin fluorescence signals. This experiment shows the value of the relative furanocoumarin fluorescence. By only considering the absolute fluorescence signals, the optimal conditions for priming seem to be the addition of SA at 48 h and following elicitation with Pep13 at 72 h. However, the relative furanocoumarin fluorescence, considering the negative controls, proves that the best conditions for priming are addition of SA at 72 h and Pep13 at 96 h.

### Comparison of two known priming-active and two priming-inactive compounds

**Fig. 7:**
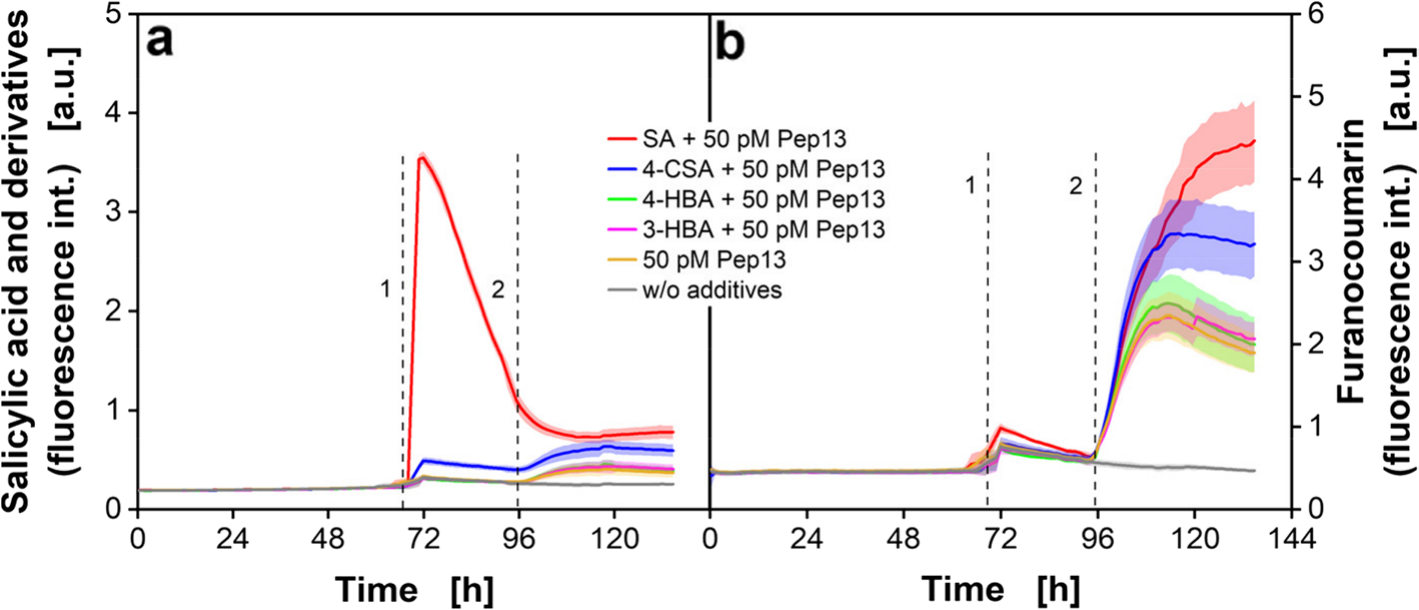
Online fluorescence intensities of parsley cell cultures treated with salicylic acid (SA) and its derivatives. Parsley cells were cultivated in 48-deep-round-well MTPs aerated with air (p_O2_ = 0.21 bar) in an in-house built BioLector. 100 µM of the priming-active compounds SA and 4-chlorosalicylic acid (4-CSA) and 100 µM of the priming-inactive compounds 4-hydroxybenzoic acid (4-HBA) and 3-hydroxybenzoic acid (3-HBA) were added after 72 h. Measurement of SA fluorescence at λ_ex_ = 295 nm and λ_em_ = 405 nm (**a**) and of furanocoumarin fluorescence at λ_ex_ = 335 nm and λ_em_ = 398 nm (**b**). Mean values were calculated from six wells. SD is indicated by colored shadows. Dashed vertical lines indicate the addition of SA or derivatives (1) and Pep13 (2). Cultivation conditions: V_L_ = 2 mL, *n* = 800 rpm, d_0_ = 3 mm and 25 °C in modified Gamborg’s B5 medium

After examining the priming procedure with SA and Pep13 in MTPs in more detail, the effect on defense priming of another priming compound and two non-priming compounds are analyzed. The aim is to show that online fluorescence measurements of furanocoumarins are suitable for identifying further priming compounds. SA and 4-CSA are used as priming-active compounds whereas 4-hydroxybenzoic acid (4-HBA) and 3-hydroxybenzoic acid (3-HBA) as priming-inactive compounds [54]. The SA and furanocoumarin fluorescence data are presented in Fig. 7.

The results for the cultures treated with SA and Pep13 are similar to the results shown in Fig. 4, Fig. 5 and Fig. 6 and demonstrate a very good reproducibility. The fluorescence properties of the three SA derivatives strongly differ from SA. The signal of SA reaches significantly higher levels (Fig. 7a, red curve) than the other compounds (Fig. 7a, blue, green and magenta curve). In Fig. 7b, an apparent increase in furanocoumarin fluorescence can be seen for SA (Fig. 7b, red curve) as well as for 4-CSA (Fig. 7b, blue curve). The online furanocoumarin fluorescence signal of both substances confirms their reported priming properties [50, 54]. The priming with SA leads to a higher production of furanocoumarins than 4-CSA. 4-HBA and 3-HBA lead to a similar furanocoumarin fluorescence increase as the negative control with only Pep13 addition (Fig. 7b, green, pink and brown curve, respectively) and confirm the priming-noninducing characteristic of 4-HBA and 3-HBA. The results are consistent with data from the literature and proof the suitability of this method to detect defense priming. In a previous study, the four substances were examined for their priming properties based on the OTR measured in shake flasks [17]. The results show that online furanocoumarin fluorescence measurements are suited to distinguishing compounds that are active at priming and those inactive at defense priming.

## Conclusions

We here reported the scale-down of studies of defense priming in parsley cell cultures from shake flasks to MTPs. The scale-down was validated by comparison of the OTR in shake flasks and the OTR, as calculated from DOT measurement in MTPs. We showed that online monitoring of the OTR in MTPs via DOT provides a unique opportunity to spot priming compounds. Furthermore, OTR monitoring was combined with fluorescence measurement of SA and furanocoumarins. The combination provided additional, valuable insight into the dynamics of priming experiments. We demonstrated that in the MTPs, low SA concentration already cause a readily-detectable priming response. We also showed that multiple combinations and times of treatment with SA and Pep13 induce successful priming. The validity of identifying priming-activating compounds based on furanocoumarin fluorescence was demonstrated with SA, 4-CSA, 4-HBA and 3-HBA. Thus, this study presents two options of high-throughput screening systems, the online DOT measurements and the online furanocoumarin fluorescence measurements, to identify and analyze the effect of known and candidate priming compounds. The non-specific method for detecting priming compounds via the OTR has the advantage of being applicable for cell suspension cultures and whole plants of species other than parsley.

## Materials and methods

### Media and solutions

Media were prepared as described previously: Parsley cell suspension was cultivated in modified Gamborg’s B5 medium. The micro- and macro-elements, including vitamins, were purchased from DUCHEFA BIOCHEMIE B.V, Haarlem, the Netherlands. The medium was supplemented with 20 g/L sucrose, 20 mg/L 2,4-dichlorophenoxyacetic acid, and 250 mg/L magnesium sulfate heptahydrate. 1 M potassium hydroxide was used to adjust the pH value to 5.5 [17]. Priming compounds were purchased at Sigma-Aldrich Co. LLC. Priming-active and priming-inactive compounds SA, 4-CSA, 4-HBA or 3-HBA were dissolved in distilled water to obtain a stock solution of 10 mM. The pH value of the stock solution was adjusted to 5.5 with a 1 M potassium hydroxide solution. The compounds were aliquoted and stored at − 20 °C. Pep13 purchased from Thermo Fisher Scientific GmbH, Germany, was dissolved in water to obtain a 5 nM stock solution aliquoted into 1.5 mL microfuge tubes and stored at -20 °C.

### Shake flask and MTP cultivation

The liquid culture was prepared from a callus every 3–4 months in modified Gamborg’s B5 medium [55] to obtain parsley (*Petroselinum crispum*) suspension cell cultures. The suspension culture was cultivated in the dark at 90 rpm in 500 mL baffled shake flasks with filling volumes of 30 mL to 40 mL, a shaking diameter d_0_ of 5 cm and 25 °C [17, 55]. The described procedure was repeated every seven days, and the parsley cells used for further experiments.

Shake flask experiments were conducted in an in-house built respiration activity monitoring system (RAMOS) device [15, 16]. Custom made versions of the RAMOS device can be acquired from Kühner AG (Birsfelden, Switzerland) or HiTec Zang GmbH (Herzogenrath, Germany). RAMOS shake flasks without baffles were used with a filling volume (V_L_) of 50 mL. The cultures consisted of one part parsley cell cultures and four parts fresh modified Gamborg’s B5 medium. The cultivations were performed in the dark with a shaking frequency (n) of 180 rpm and a shaking diameter (d_0_) of 5 cm at 25 °C.

MTP experiments were conducted in sterile 48-deep-round-well MTPs (MTP-R48-BOH, Beckman Coulter GmbH, Baesweiler, Germany) using a custom made BioLector device (Beckman Coulter GmbH, Germany) or an in-house built device with a connected Spectrofluorometer (Fluoromax-4, HORIBA Jobin Yvon GmbH, Unterhaching, Germany) [21, 22]. The MTP cultivations were performed in modified Gamborg’s B5 Medium with V_L_ = 2 mL, n = 600 rpm or 800 rpm and d_0_ = 3 mm at 25 °C in the dark. The oxygen level in the inlet gas was set to p_O2_ = 0.21 bar, if not stated otherwise. For the experiments with reduced oxygen content, a mixture of nitrogen and air was used to adjust the oxygen level to p_O2_ = 0.15 bar. The flow of nitrogen and air to the aeration unit was regulated by two mass flow controllers (Brooks Instruments, Ede, The Netherlands). A constant volume flow of 60 mL/min was adjusted.

### Hydrodynamics in a well

A miniature CCD-camera (XC-777AP, Sony) was attached to a shaking platform to record the shaking behavior of parsley cell suspension in a single well of a clear 48-deep-round-well MTP. The clear MTP was kindly provided by m2p-labs (now Beckman Coulter) and had the same geometrics as the 48-deep-round-well plates for DOT and fluorescence measurements. The pictures were taken with a filling volume of 2 mL, an inner well diameter (D_w_) of 12.4 mm and a shaking diameter (d_0_) = 3 mm [26, 30]. Images of parsley cell suspension cultures were taken for shaking frequencies (n) from 0 to 700 rpm for evaluation of mixing behaviour. The critical shaking frequency (n_crit_) [rpm] was calculated according to Hermann et al. [26] (see Eq. 1), taking into account the shaking diameter (d_0_) [m] of 0.003 m, the well diameter (D_w_) [m] of 0.00124 m and the filling volume (V_L_) of 0.002 L. The surface tension (σ) [N/m] of the modified Gamborg’s B5 medium was determined by the Wilhelmy plate method (tensiometer from Fa. Lemke Parter, Kaarst, Germany; platin plate from Krüss GmbH, Hamburg, Germany). The mean surface tension of the modified Gamborg’s B5 medium was 69.00 ± 1.03 N/m (*n* = 3). To estimate the liquid density, the modified Gamborg’s B5 medium was assumed to act as an ideal mixture without intermolecular interactions and without solving effects of the medium. The weight of each component in 1 L H_2_O was added up and combined with the weight of 1 L H_2_O. The liquid density (ρL) was 1023.4 kg/m^3^.

1 $${n}_{crit}=\sqrt{\frac{\sigma \cdot {D}_{w}}{4\cdot \pi \cdot {V}_{L}\cdot {\rho }_{L}\cdot {d}_{0}}}$$

### Standard priming procedure

The standard priming procedure applied in this study is based on the method developed by Kauss et al. (1992) [10]. This method was successfully used numerous times, to detect priming-active compounds [11, 13, 14, 51]. The parsley suspension cell culture were treated with 2,6-dichloroisonicotinic acid (DCIA) or SA as priming-active compound. A fungal elicitor was added after 96 h, followed by the fluorescence measurement of the furanocoumarins 24 h later. Kauss et al. tested varying DCIA addition times and concentrations. They also analyzed several time points of measuring the furanocoumarin fluorescence. In the present study, this priming method was adopted as follows. The adopted standard priming procedure included the addition of 100 µM of a priming-active compound or a priming-inactive compound as (salicylic acid (SA), 4-chlorosalicylic acid (4-CSA)), 4-hydroxybenzoic acid (4-HBA) and 3-hydroxybenzoic acid (3-HBA) after 72 h. The priming-active and priming-inactive properties of SA, 4-CSA, 4-HBA or 3-HBA were shown before [17, 54]. 24 h after the SA, 4-CSA, 4-HBA or 3-HBA treatment, 50 pM of the elicitor Pep13 were added to the culture. In experiments with varying concentrations or times of addition, the exact doses are specified in each case's results section and the captions of the corresponding figure. In shake flask experiments, the added volume per addition was 1 mL. In MTP experiments, 40 µL of the respective substance were added. A sample was taken after 120 h to determine the furanocoumarin fluorescence intensity in experiments without an online fluorescence signal.

### Determination of the DOT and OTR

RAMOS enables the quasi-continuous measurement of the oxygen partial pressure in the headspace of a shake flask. From this measurement, the OTR can be calculated. For the determination of the OTR in MTPs, the dissolved oxygen tension (DOT) was determined via online fluorescence measurements. For this purpose, each well of the MTP was equipped with an oxygen sensor spot. Two different sensor spots, the PreSens spots, already integrated into the MTP from Beckman Coulter and spots from PyroScience (OXSP5, PyroScience GmbH, Aachen, Germany), were used. The wavelength pair for DOT measurement in MTPs with PreSens sensor spots (MTP-R48-BOH, Beckman Coulter GmbH (formerly m2p-labs), Baesweiler, Germany) was λ_ex_ = 520 nm and λ_em_ = 600 nm and the experiments were conducted using a custom made BioLector device from Beckman Coulter. The wavelength pair for DOT measurements in MTPs with PyroScience spots was λ_ex_ = 610 nm and λ_em_ = 760 nm, and the experiments were conducted using the in-house built BioLector. For calibration, wells were filled with 2 mL modified Gamborg’s B5 medium. Oxygen partial pressure of p_O2_ = 0 bar and p_O2_ = 0.21 bar, or p_O2_ = 0.15 bar were adjusted. The Stern–Volmer relationship allowed the calculation of the oxygen partial pressure from fluorescence intensities using the following equation:

2 $$\frac{f_0}f=1+k_{sv}\cdot DOT$$

*f*_0_ is the fluorescence intensity in the absence of oxygen, *f* is the fluorescence intensity for given content of the quencher oxygen DOT [%], and k_sv_ is the Stern–Volmer constant. With the obtained DOT, the OTR can be calculated (OTR_calc_) using the correlation from Wewetzer et al. [23].

3 $${OTR}_{calc}=k_La\cdot L_{O2}\cdot\left({pO}_2^{gas}-\left(\frac{DOT}{100}\right)\cdot{pO}_2^{cal}\right)$$

k_L_a [1/h] is the volumetric oxygen mass transfer coefficient, L_O2_ [mol/L/bar] is the oxygen solubility, p_O2_^gas^ [bar] is the partial pressure of oxygen in the gaseous phase, and p_O2_^cal^ [bar] is the partial pressure during the calibration of the DOT. Oxygen solubility was calculated to be 1.22 mmol/L/bar as described before [32–34]. The k_L_a in the MTPs with V_L_ = 2 mL, *n* = 600 rpm and d_0_ = 3 mm at 25 °C was approximated to be 37 1/h by the smallest sum of square errors between OTR and OTR_calc_ vectors from both shake flask and MTP [23, 36]. The calculation of the OTR was performed under the assumption of a constant oxygen partial pressure p_O2_^gas^ of 0.21 bar. The mamximum oxygen transfer capacity (OTR_max_), used to ensure oxygen-unlimited conditions in MTP cultivations, was calculated with the following equation:

4 $${OTR}_{max}=k_La\cdot L_{O2}\cdot{pO}_2^{gas}$$

### Normalization of DOT and OTR data

The parsley cell cultures differ in their respiration activity even before the addition of SA and Pep13. Since differences after priming and the addition of the elicitor are investigated, the DOT and OTR values are normalized on the y-axis before the addition of SA at 72 h cultivation time. Hence, the mean value of all cultures between 68 and 70 h cultivation time was calculated. Subsequently, the difference between the mean values of all cultures and the mean value of the individual cultures in the given time was calculated. This difference was then subtracted from all values of each culture in the entire cultivation period. As a result, curves were aligned on top of each other before SA was added. Additional file 3 shows an example of the normalization for the curves shown in Fig. 3e.

### Fluorescence measurements of SA and furanocoumarins

In shake flask cultivations, the fluorescence of furanocoumarins was measured offline in cuvettes. The culture broth was harvested after 120 h and centrifuged at 4000 rpm for 10 min at 4 °C. Subsequently, 3 mL of the supernatant were transferred into a quartz cuvette (10 × 10 mm Suprasil quartz, Hellma GmbH & Co. KG, Müllheim, Germany) and measured with a fluorescence spectrometer (Fluoromax-4, HORIBA Jobin Yvon GmbH, Unterhaching, Germany) with an excitation wavelength of λ_ex_ = 335 nm and an emission wavelength of λ_em_ = 398 nm [11, 13, 17]. The online fluorescence measurements of furanocoumarins and salicylic acid in MTPs were performed at *n* = 600 rpm using the in-house built BioLector with an attached fluorescence spectrometer. The furanocoumarin fluorescence was measured at λ_ex_ = 335 nm and λ_em_ = 398 nm, and the salicylic acid fluorescence at λ_ex_ = 295 nm and λ_em_ = 405 nm. Two different types of fluorescence data are shown in this paper: On the one hand, the absolute values of fluorescence (raw data) of salicylic acid or furanocoumarin ${f}_{x}$, and on the other hand, the calculated relative furanocoumarin fluorescence ${f}_{{x}_{rel}}$. To calculate the corrected furanocoumarin fluorescence signals ${f}_{{x}_{c}}$, the total furanocoumarin fluorescence signal of the SA and Pep13 treated cultures was subtracted by the total furanocoumarin fluorescence signal of the untreated culture without additives f_w/o additives_.

5 $${f}_{{x}_{c}}{= f}_{x}-{f}_{w/o additives}$$

To gain the relative furanocoumarin fluorescence ${(f}_{{x}_{rel}}$) in %, the sum of the corrected fluorescence intensities of the cultures treated exclusively with SA or Pep13 was set to 100%. The corrected fluorescence intensities of SA and Pep13 treated specimens were put in relation.

6 $$f_{x_{rel}}=\frac{f_{x_c}}{f_{{PrimingCompound}_c}+f_{{Pep13}_c}}\cdot100$$

### Calculation of errors

The number of replicates is different, depending on the experiment. Each experiment was conducted with at least three replicates and with a maximum of ten replicates. The only exceptions are the OTR curves from the shake flask experiments in Fig. 2 and Fig. 3a. Here, no further replicates were carried out, as this experimental conditions had already been investigated several times and published in a preceding paper [17]. The OTRs of this shake flask experiments in Figs. 2 and 3a are in agreement with the published data, which ensures the reliability of the results. The number of relevant replicates for error calculation for each experiment is specified in the figure captions. The following equation was used to calculate the standard deviation:

7 $$s\left(x\right)=\sqrt{\frac{1}{N-1}\sum_{i=1}^{N}{({x}_{i}-x)}^{2}}$$

The standard error propagation for calculating the standard deviation of the relative fluorescence intensities was calculated by Eq. 8.

8 $$s_f=\sqrt{\left(\frac{\partial_f}{\partial_x}\right)^2\cdot s_x^2+\left(\frac{\partial_f}{\partial_y}\right)^2\cdot s_y^2+\left(\frac{\partial_f}{\partial_z}\right)^2\cdot s_z^2+\cdot\cdot\cdot}$$

### Statistical analysis

To determine whether or not the OTR of the Pep13-treated cultures and the SA- and Pep13-treated experiments analyzed by PyroScience and PreSens sensor spots differs significantly, a student´s t-test was performed [56]. The difference in OTR values was validated by a 5% significance level.

## Supplementary Information

**Additional file 1. **Figure S1, Figure S2, Figure S3.

## Data Availability

The datasets used and analyzed during the current study are available from the corresponding author on reasonable request.

## Abbreviations

3-HBA: 3-Hydroxybenzoic acid
4-CSA: 4-Chlorosalicylic acid
4-HBA: 4-Hydroxybenzoic acid
d_0_: Shaking diameter [mm]
DOT: Dissolved oxygen tension [%]
D_w_: Inner well diameter [mm]
*f*: Fluorescence intensity [-]
*f*_0_: Fluorescence intensity in the absence of oxygen [-]
f_w/o additives_: Total furanocoumarin fluorescence signal of the untreated culture without additives [-]
${f}_{{x}_{c}}$: Corrected furanocoumarin fluorescence signals [-]
${f}_{{x}_{rel}}$: Calculated relative furanocoumarin fluorescence [%]
k_L_a: Volumetric mass transfer coefficient [1/h]
k_sv_: Stern–Volmer constant [1/%]
L_O2_: Oxygen solubility [mol/L/bar]
MTP: Microtiter plate
n: Shaking frequency [rpm]
n_crit_: Critical shaking frequency [rpm]
OTR: Oxygen transfer rate [mmol/L/h]
OTR_calc_: Calculated oxygen transfer rate [mmol/L/h]
OTR_max_: Maximal oxygen transfer capacity [mmol/L/h]
p_O2_: Oxygen partial pressure [bar]
_pO2_^cal^: Oxygen partial pressure during the calibration of the DOT [bar]
p_O2_^gas^: Oxygen partial pressure of oxygen in the gaseous phase [bar]
Q_O2_: Content of the quencher oxygen [%]
RAMOS: Respiration activity monitoring system
SA: Salicylic acid
SAR: Systemic acquired resistance
V_L_: Filling volume [mL]
ρL: Liquid density [kg/m^3^]
σ: Surface tension [N/m]
λ_em_: Emission wavelength [nm]
λ_ex_: Excitation wavelength [nm
