# Supplementary material for: Spotting priming-active compounds using parsley cell cultures in microtiter plates
Source: BMC Plant Biol. 2023 Feb 2;23:72. doi: 10.1186/s12870-023-04043-y (PMC9893529; doi:10.1186/s12870-023-04043-y)
Supplement: Supplementary file 1 — Additional file 1. Figure S1, Figure S2, Figure S3. [file 12870_2023_4043_MOESM1_ESM.docx]

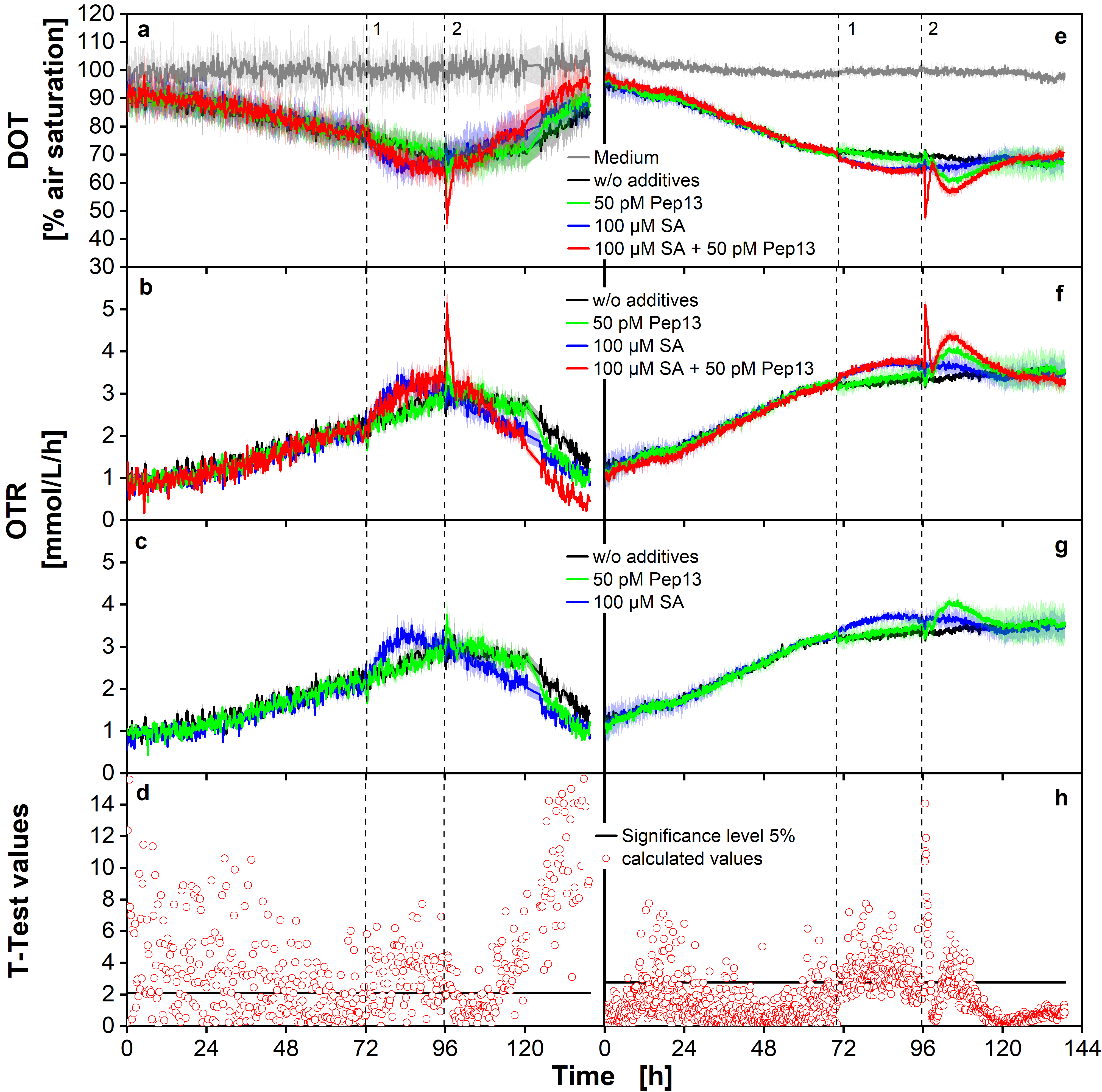


Additional file 1 DOT and calculated OTR of parsley cell cultures measured with two different sensor spot systems. Parsley cell cultures aerated with air (p_O2_ = 0.21 bar) were cultivated in 48-deep-round-well MTPs with PreSens sensor spots using a custom made industrial BioLector device (**a**) and in 48-deep-round-well MTPs with PyroScience sensor spots using an in-house built BioLector (**e**). The OTR in **b** and **f** was calculated from the DOT (**a** and **e**) according to Equation 3. Since the red curve in **b** and **f** partly covers the other curves, the graphs **b** and **f** are shown again without the red curve in **c** and **g**, respectively. Dashed vertical lines indicate the addition of salicylic acid (SA) (1) and Pep13 (2). Mean values were calculated from 10 wells in **a** and **b** and from three wells in **e** and **f**, respectively. The SD in **a**, **b**, **c**, **e**, **f** and **g** is indicated by colored shadows. The data were normalized as described in Material and Methods. MTP cultivation conditions: V_L_ = 2 ml, n = 600 rpm, d_0_ = 3 mm and 25 °C in modified Gamborg’s B5 medium. **d** and **h** show the T-test values for the comparison of the Pep13 treated cultures (**b**,**c**, **f** and **g** green line) and the SA and Pep13 treated experiments (**b** and **f** red line). The black dashed horizontal lines in **d** and **h** show the 5% quantile of the t_n_-distribution.


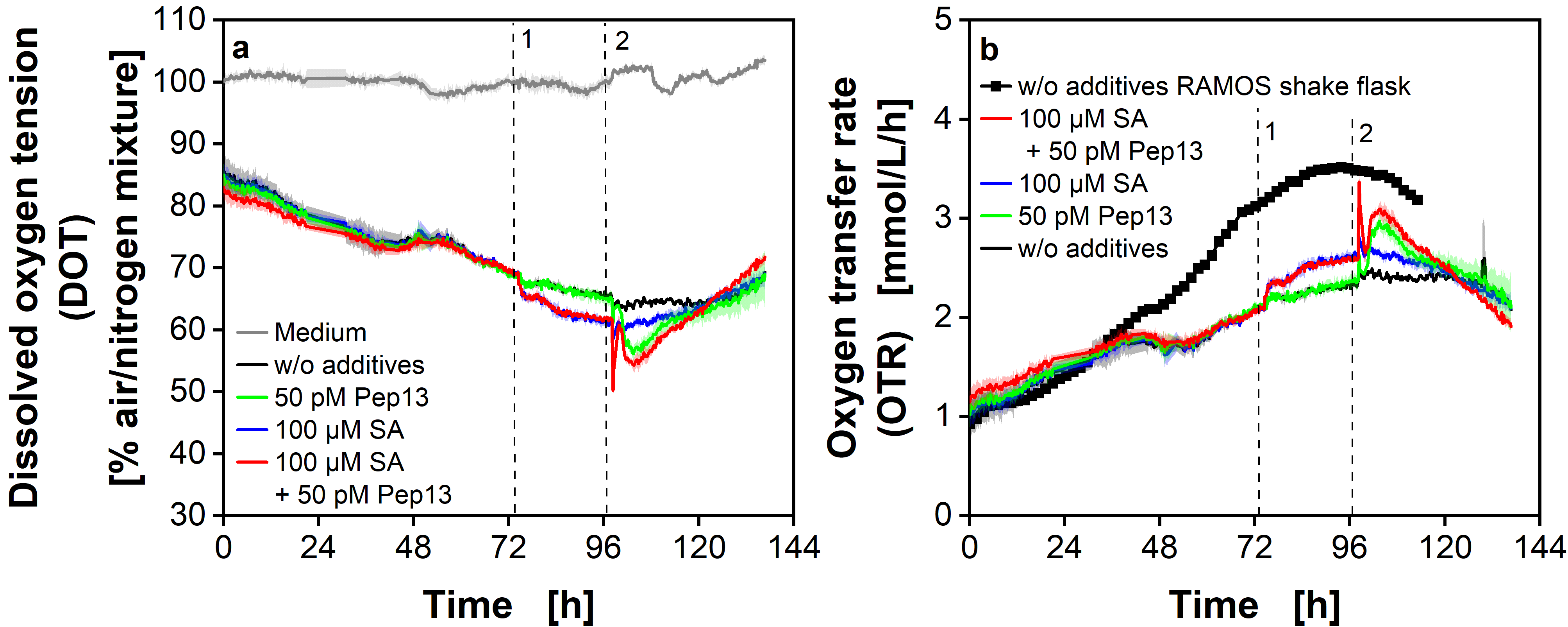


Additional file 2 DOT and OTR of parsley cell cultures in a MTP aerated with an air/nitrogen mixture. Parsley cell cultures aerated with an air/nitrogen mixture (partial pressure of oxygen in supplied gas mixture p_O2_ = 0.15 bar) were cultivated in 48-deep-round-well MTPs. PyroScience sensor spots were used to measure the DOT with an in-house built BioLector (**a**). The OTR was calculated from the DOT according to Equation 3 and is shown in **b**, compared to a reference cultivation from shake flasks. Dashed vertical lines indicate the addition of salicylic acid (SA) (1) and Pep13 (2). MTP cultivation conditions: V_L_ = 2 ml, n = 600 rpm, d_0_ = 3 mm and 25 °C in modified Gamborg’s B5 medium. Mean values were calculated from three wells and the SD is indicated by colored shadows. The data were normalized as described in Material and Methods. Parameters for OTR calculation: k_L_a = 37 1/h, L_O2_ = 1.22 mmol/L/bar, p_O2_ = 0.15 bar. Shake flask cultivation conditions p_O2_ = 0.21 bar, 250 mL RAMOS flasks, V_L_ = 50 mL, n = 180 rpm, d_0_ = 50 mm and 25 °C.


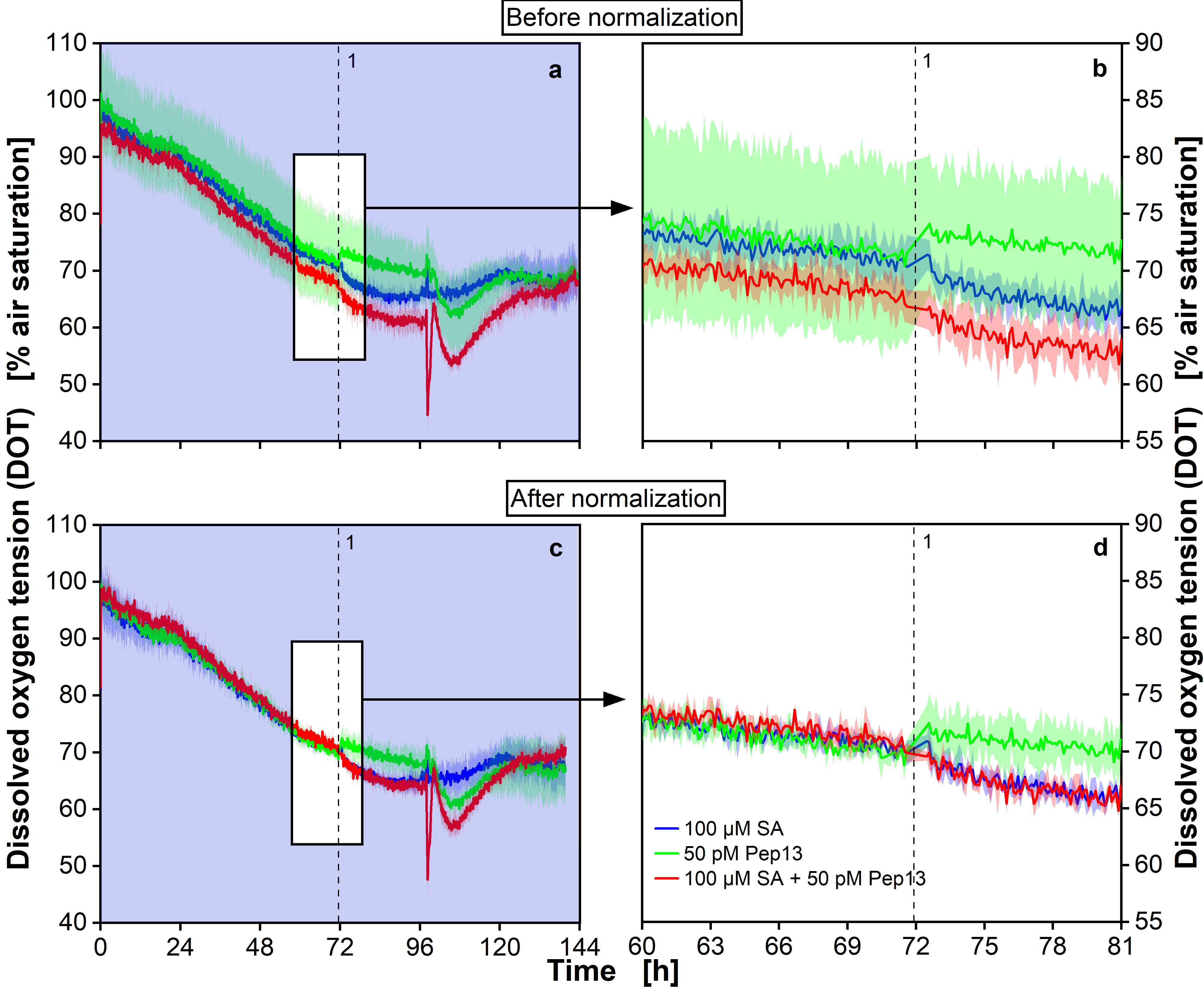
 Additional file 3 DOT of parsley cell cultures before (**a** and **b**) and after (**c** and **d**) normalization. Normalization was performed before the addition of salicylic acid (SA) after 72 h (Material and Methods: Normalization of DOT and OTR data). The data are drawn from the cultivation shown in Fig. 3**e.** In **a** and **c** inserts are depicted, which are enlarged in **b** and **d**. In **c** and **d** it is shown, how curves are aligned on top of each other after normalization. Parsley cell cultures were cultivated in 48-deep-round-well MTPs. 100 µM SA was added after 72 h and 50 pM Pep13 after 96 h. PyroScience sensor spots were used to measure the DOT with an in-house built BioLector. The dashed vertical lines indicate the addition of SA after 72 h. MTP cultivation conditions: V_L_ = 2 ml, n = 600 rpm, d_0_ = 3 mm and 25 °C in modified Gamborg’s B5 medium. Mean values were calculated from three wells. The standard deviation is indicated by colored shadows.
